# Effects of Corticosteroid Treatment and Antigen Avoidance in a Large Hypersensitivity Pneumonitis Cohort: A Single-Centre Cohort Study

**DOI:** 10.3390/jcm8010014

**Published:** 2018-12-21

**Authors:** Laurens J. De Sadeleer, Frederik Hermans, Els De Dycker, Jonas Yserbyt, Johny A. Verschakelen, Eric K. Verbeken, Geert M. Verleden, Wim A. Wuyts

**Affiliations:** 1 Department of Respiratory Diseases, Unit for interstitial lung diseases, University Hospitals Leuven, Leuven B-3000, Belgium; frederik.hermans@yahoo.com (F.H.); els.dedycker@uzleuven.be (E.D.D.); jonas.yserbyt@uzleuven.be (J.Y.); geert.verleden@uzleuven.be (G.M.V.); wim.wuyts@uzleuven.be (W.A.W.); 2 Laboratory of Respiratory Diseases, Department of Chronic Diseases, Metabolism & Ageing (CHROMETA), KU Leuven, Leuven B-3000, Belgium; 3 Department of Radiology, University Hospitals Leuven, Leuven B-3000, Belgium; johny.verschakelen@uzleuven.be; 4 Department of Pathology, University Hospitals Leuven, Leuven B-3000, Belgium; eric.verbeken@uzleuven.be

**Keywords:** hypersensitivity pneumonitis, treatment, mixed models

## Abstract

Background: Although the third most frequent interstitial lung disease, hypersensitivity pneumonitis (HP) remains an enigmatic disease without clear diagnostic and therapeutic guidelines. We assessed the effect of the commonly used therapeutic interventions (i.e. exposure avoidance and corticosteroid treatment) in an HP cohort. Methods: We collected clinical data of all HP patients followed at our centre between January 1, 2005, and December 31, 2016. HP patients were stratified according to the presence of fibrosis on chest CT. Survival was analysed using the multivariate Cox proportional hazards model. Forced vital capacity (percent predicted, FVC%) and diffusing capacity of the lung for carbon monoxide (percent predicted, DLCO%) evolution were analysed using linear mixed-effect models. Results: Two hundred and two HP patients were identified: 93 non-fibrotic HP (nfHP) and 109 fibrotic HP (fHP), experiencing a monthly FVC% decline before treatment of 0.93% and 0.56%, respectively. While nfHP had an excellent survival, fHP patients experienced a median survival of 9.2 years. Corticosteroid treatment and exposure avoidance did not result in survival differences. Although nfHP patients showed FVC% and DLCO% increase after corticosteroid initiation, no therapeutic effect was seen in fHP patients. FVC% and DLCO% increased in nfHP patients after exposure avoidance, while a positive numerical trend was seen for FVC% after exposure avoidance in fHP patients (*p* = 0.15). Conclusions: nfHP patients experienced an excellent survival with good therapeutic effect on pulmonary function tests with both corticosteroid initiation as well as antigen avoidance. In contrast, fHP patients experienced a dismal prognosis (median survival of 9.2 years) without any therapeutic effect of corticosteroid treatment. Whether antigen avoidance is useful in fHP patients is still unclear.

## 1. Introduction

Hypersensitivity pneumonitis (HP) is the third most frequent interstitial lung disease (ILD) [1], characterized by an aberrant immunological reaction upon a (mainly organic) antigen, which can lead to a profibrotic response [2,3]. Therapeutic approach consists mainly of antigen avoidance and immunosuppressive treatment; however, evidence to support this strategy is very limited, based on data in acute HP [4,5] and limited retrospective series [6,7].

Stratification of HP patients is classically based on symptom chronicity as well as presenting symptoms [8,9,10]. Conversely, recent literature suggested stratification based on radiologic and histopathologic features, would correlate more with the natural history of the disease [11,12]: HP patients with fibrosis have a prognosis of only 5–8 years—far worse compared to HP patients without fibrosis [13,14,15,16,17,18]. Some reports have also suggested that patients with honeycombing experience outcomes similar to Idiopathic Pulmonary Fibrosis (IPF) patients with honeycombing [12]. 

For this study, we sought to investigate the therapeutic effect on survival and pulmonary function test evolution of both antigen avoidance and corticosteroid initiation in both non-fibrotic HP (nfHP) and fibrotic HP (fHP) with the hypothesis that treatment effects would be different between these two subgroups.

## 2. Experimental Section

### 2.1. Patient Selection and Data Collection

All patients presenting at the University Hospitals Leuven between January 1, 2005, and December 31, 2016, with a diagnosis of HP were included. The HP diagnosis was validated using one of the following criteria, in line with recent literature [8,9,19,20]:

- Combination of compatible symptoms (dyspnoea and/or cough and/or symptoms with onset shortly after exposure (max 4–6 h) and/or recurrent viral symptoms), suggestive CT findings (extensive ground glass opacities (GGO) and/or centrilobular noduli and/or peribronchovascular fibrosis and/or upper lobe predominant fibrosis and/or airtrapping) and—if biopsy was performed—suggestive pathology data (presence of granuloma’s and/or giant cells and/or a bronchocentric inflammatory infiltrate and/or bronchocentric fibrosis and/or fibrosis with a mixed bronchocentric/usual interstitial pneumonia (UIP)-like pattern)
- Combination of compatible symptoms (cf supra) and suggestive CT findings (cf supra) and compatible pathology findings (any fibrosis and no other disease more probable according to the pathology report)
- Combination of compatible symptoms (cf supra) and compatible CT findings (fibrosis with a UIP-like or non-specific interstitial pneumonia (NSIP)-like pattern) and suggestive pathology findings (cf supra).

Cases not complying with one of these criteria were discussed in a multidisciplinary discussion (MDD). Reports of these MDDs can be found in Supplementary Table S1 (including reason for non-compliance and MDD’s final advice). Baseline characteristics, clinical data concerning exposure, serological test results, broncho-alveolar lavage (BAL) cell count, survival data, pulmonary function tests (PFT), and high resolution computed tomography (HRCT) at diagnosis were retrieved from the patient files. Patients were followed up until April 1, 2018. 

### 2.2. Outcome

Patients were divided in two HP subgroups (nfHP and fHP), based on HRCT findings [8,11,12]: fibrosis was defined as the presence of extensive reticulation, traction bronchiectasis, honeycombing on HRCT, or a combination of these. HP patients without fibrosis on HRCT were included in the nfHP group, HP patients with fibrosis were included in the fHP group. Differences in baseline characteristics, survival, and PFT evolution were assessed between the two groups. The effect of corticosteroid therapy initiation and antigen avoidance on survival and Forced vital capacity (percent predicted, FVC%) and diffusing capacity of the lung for carbon monoxide (percent predicted, DLCO%) decline were assessed. 

### 2.3. Statistical Analysis

Baseline characteristics: Continuous variables were analysed using Student’s *t*-tests or Mann Whitney U-test where appropriate. For discrete variables, chi square tests and Fisher’s exact tests were used, where appropriate. 

Survival analysis: outcome was based on 10-year survival. Data were displayed as Kaplan Meier curves and analysed using Cox proportional hazards models. Patients who underwent lung transplantation were censored at the day of transplantation. In multivariate analyses, we corrected for age, gender, and baseline FVC%. All multivariate analyses are shown in Supplementary Table S2. 

PFT evolution: evolution of PFT was analysed with mixed effect models, using FVC% and DLCO% as outcome measurements (in separate analyses) with subject as a random effect and time, age, and gender were accounted for as fixed effect. 

Corticosteroid treatment analysis: corticosteroid use was accounted for as fixed effect, both with and without time-varying covariate. Exposure status was corrected for as fixed effect and immunosuppression use as a time-varying covariate. 

Antigen avoidance analysis: exposure status and corticosteroid use were corrected for with and without time-varying covariate and immunosuppression use as time-varying covariate. 

Informed consent and ethical approval were obtained at the University Hospitals Leuven ethical commission. Analyses were conducted according to ‘strengthening the reporting of observational studies in epidemiology’ (STROBE) guidelines [21]. Statistical analyses were performed in R (version 3.3.1, R Project for Statistical Computing, Vienna, Austria). More detailed information concerning outcome measurements and statistical analysis can be found in Supplementary Appendix S1.

## 3. Results

### 3.1. Diagnostic Categories and Considerations

**Figure 1:**
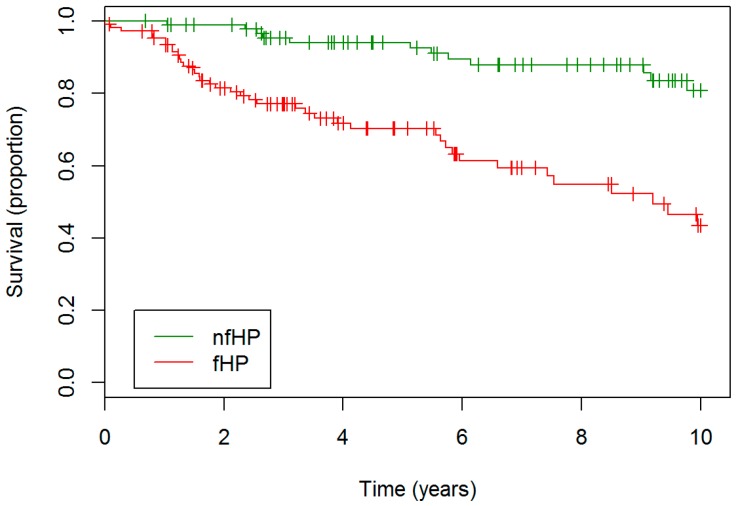
**Survival nfHP and fHP patients**. fHP patients experienced a worse outcome compared to nfHP patients. Definition of abbreviation: nfHP, non-fibrotic hypersensitivity pneumonitis, fHP, fibrotic chronic hypersensitivity pneumonitis

Two hundred and two patients met the inclusion criteria: 93 were classified as nfHP and 109 as fHP. Overview of cohort formation is presented in Supplementary Figure S1. fHP patients were older, had lower baseline FVC% and DLCO%, and lower BAL lymphocytosis (Table 1). fHP patients more often had unknown exposure and were treated more often with second line immunosuppressive agent. Median survival of fHP patients was 9.2 years, whereas the median survival of nfHP was not reached. fHP patients experienced a worse outcome compared to nfHP (hazard ratio (HR) 4.35, confidence interval (CI) 2.22–8.33, *p* < 0.001), as presented in Figure 1. 

PFT data were collected from the 202 included patients. In total, 1251 and 1604 PFTs were collected from the clinical files of nfHP and fHP patients, respectively. Throughout the first year of their follow-up (before treatment initiation), there was no difference in FVC% per month between nfHP or fHP groups (0.93% and 0.56%, respectively; *p* = 0.75).

### 3.2. Corticosteroid Treatment

Corticosteroids were used in 67 (79%) nfHP patients and 82 (80%) fHP cases. In 8 (10%)-nfHP patients and 24 (25%) fHP patients an additional immunosuppressive agent was used. Baseline characteristics according to corticosteroid treatment status and 2nd line immunosuppressive treatment status can be found in Supplementary Table S3–S4. Throughout the entire cohort (both nfHP and fHP patients), patients treated with corticosteroid therapy had lower baseline FVC% (73% vs. 91%, *p* < 0.001) and DLCO% (46% vs. 65%, *p* < 0.001), were more often female (76% vs. 55%, *p* = 0.03), and were prescribed more often second line immunosuppressive agents (22% vs. 3%, *p* = 0.006). 

**Figure 2:**
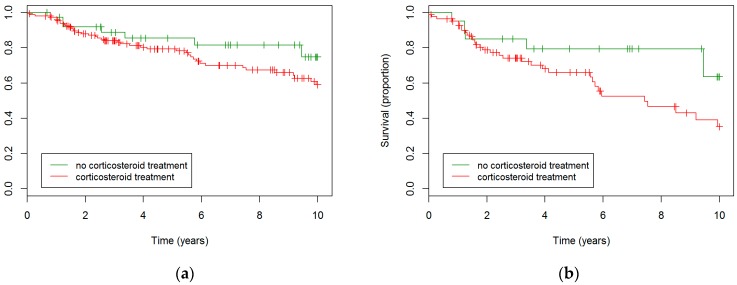
**Survival in corticosteroid-treated patients compared to never-treated patients**. (**a**) Kaplan Meier based on the entire cohort. No statistically significant differences were observed. (**b**) Kaplan Meier based on the fHP subgroup. A trend towards worse survival in the treated group was observed (*p* = 0.096).

Corticosteroid treatment did not result in different survival rates in the entire cohort (Figure 2a). A trend towards worse survival in the treated group was seen in the fHP subgroup (HR 2.2, *p* = 0.096), as displayed in Figure 2b. This trend disappeared in multivariate analysis correcting for age, gender, and baseline FVC%; however, no sign towards better survival was observed in the treated group either.

**Figure 3:**
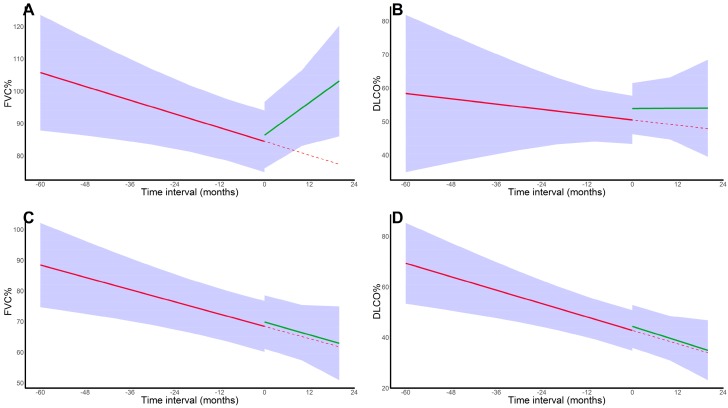
**Pulmonary function test evolution in corticosteroid-treated patients**. (**A**). FVC% trajectory in nfHP patients; (**B**). DLCO% trajectory in nfHP patients; (**C**). FVC% trajectory in fHP patients; (**D**). DLCO% trajectory in fHP patients. For FVC%, a positive effect of corticosteroid initiation was seen in nfHP, whereas this effect was absent in the fHP group. For DLCO%, no impact on decline was observed however, nfHP patients experienced an immediate numerical increase in DLCO% after treatment initiation. The solid red line represents the mixed effect model estimates before corticosteroid initiation, the solid green line represents the mixed effect model estimates after corticosteroid initiation and the dotted red line represents the pre-treatment trend. Subject was corrected for as a random effect, both with random intercept and (independent) random slope. Fixed effects were time, age, gender, ever smoker status, and immunosuppression use. Definition of abbreviation: nfHP, non-fibrotic hypersensitivity pneumonitis; fHP, fibrotic chronic hypersensitivity pneumonitis.

In nfHP, corticosteroid initiation resulted in a reversal from a monthly FVC% decline of 0.35% to an FVC% increase of 0.84% (*p* < 0.001). For DLCO% decline, no significant differences were observed (*p* = 0.43); however, nfHP patients showed a trend towards an immediate DLCO% increase of 3.38% after corticoid treatment initiation (*p* = 0.081). In contrast, no significant changes were observed for fHP (FVC%: *p* = 0.96, DLCO%: *p* = 0.59). Results are shown in Figure 3. For fHP, no differences were observed between patients treated <6 months vs. >6 months and with <40 mg prednisolone-equivalent vs. ≥40mg prednisolone-equivalent (as maximal dose), in terms of baseline characteristics, survival (Supplementary Figures S2–S3), or PFT evolution. Finally, similar results were observed in an analysis confined to fHP patients referred to our centre before corticosteroid initiation (FVC%: *p* = 0.57; DLCO%: *p* = 0.41).

### 3.3. Exposure Type

The inciting agent remained unknown in 41 cases (20%): 11% of nfHP and 28% of fHP cases (*p* = 0.003). Overall, patients without known inciting agent had lower baseline FVC% (68% vs. 78%, *p* = 0.03), were more often female (65% vs. 41%, *p* = 0.009)) and more often ever-smokers (46% vs. 30%, *p* = 0.095) and showed more often signs of fibrosis (76% vs. 49%, *p* = 0.004). 

**Figure 4:**
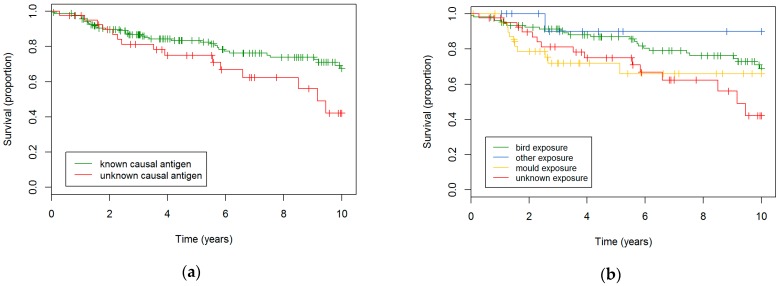
**Survival in HP patients according to exposure**. (**a**) Survival in HP patients with known and unknown antigen A trend towards worse survival in the patient group with unknown antigen was observed (HR 1.8, *p* = 0.056), which became statistically significant after multivariate correction (HR 1.8, *p* = 0.045). (**b**) Survival in HP patients based on exposure type. Bird exposure resulted in a better survival: vs. mould exposure (HR 2.0, *p* = 0.057); unknown exposure (HR 2.1, *p* = 0.029).

Throughout the entire cohort (both nfHP and fHP patients), there was a trend towards worse survival in patients without known inciting agent (HR 1.8, *p* = 0.056), that was significant after multivariate correction (HR 1.8, *p* = 0.045, Figure 4a). In the nfHP subgroup, patients with unknown antigen showed steeper FVC% decline (*p* = 0.026), which could not be replicated in the fHP cohort (*p* = 0.26). No differences in DLCO% decline were observed (nfHP: *p* = 0.59; fHP: *p* = 0.41). 

Overall, bird, mould, and other exposures were found in 53%, 20%, and 7% of patients, respectively (while 20% of patients had unknown exposure). Prevalence of exposure types differed between nfHP and fHP (*p* = 0.003), as shown in Table 2.

Bird-exposed HP patients had a better survival compared to mould-exposed patients (HR 2.0, *p* = 0.057) or patients without known exposure (HR 2.1, *p* = 0.029, Figure 4b). These results were significant after multivariate analysis for both comparisons (vs mould: HR 2.8, *p* = 0.007; vs. unknown exposure: HR 2.8, *p* = 0.009). No differences in FVC% and DLCO% evolution could be observed.

### 3.4. Exposure Avoidance

**Figure 5:**
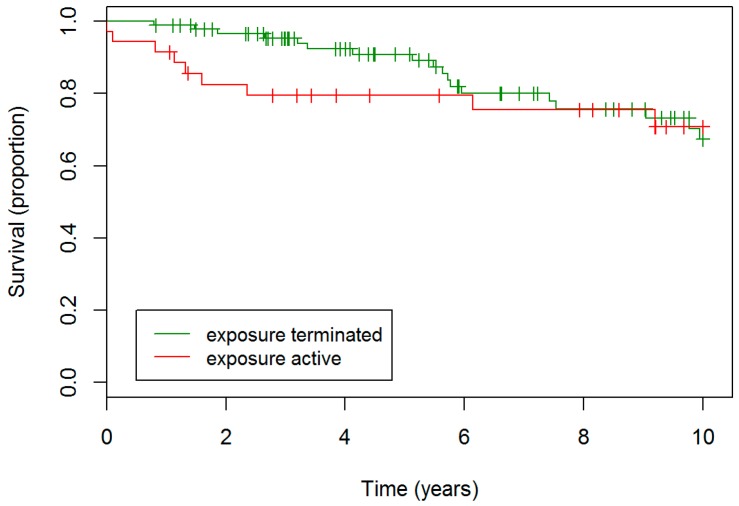
**Survival in HP patients based on active exposure throughout follow-up**. No significant differences were observed

Seventy-two percent of patients with an identifiable cause terminated the exposure. No differences in baseline characteristics were found between patients who stopped the exposure and those who continued their exposure. Rates of corticosteroid treatment (known cause: 80% vs. unknown cause: 79%, *p* = 1) and treatment with a second line immunosuppressive treatment (20% vs. 11%, *p* = 0.24) were similar; neither were survival differences found (*p* = 0.61, Figure 5). 

**Figure 6:**
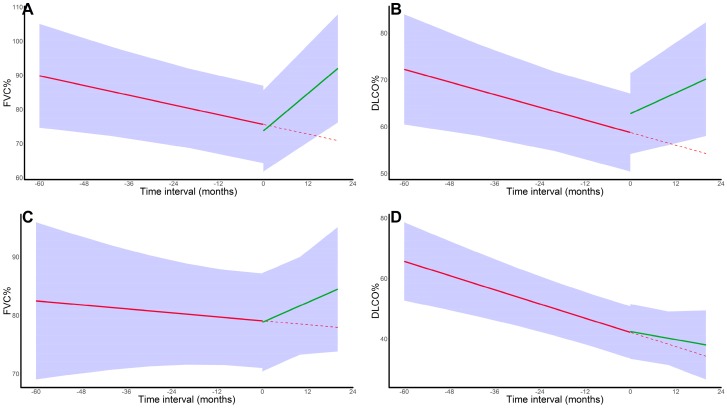
**Pulmonary function test evolution in patients who terminated the exposure**. (**A**). FVC% trajectory in nfHP patients; (**B**). DLCO% trajectory in nfHP patients; (**C**). FVC% trajectory in fHP patients; (**D**). DLCO% trajectory in fHP patients. nfHP patients experienced both an FVC% and DLCO% increase after exposure avoidance. fHP patients experienced a non-significant trend towards FVC% increase after exposure avoidance (*p* = 0.15). The solid red line represents the mixed effect model estimates before exposure termination, the solid green line represents the mixed effect model estimates after exposure termination and the dotted red line represents the pre-treatment trend. Subject was corrected for as a random effect, both with random intercept and (independent) random slope. Fixed effects were time, age, gender, ever smoker status, and immunosuppression use. Definition of abbreviation: nfHP, non-fibrotic hypersensitivity pneumonitis; fHP, fibrotic chronic hypersensitivity pneumonitis.

In nfHP patients, FVC% decline of 0.24% monthly was reversed to a 0.92% monthly FVC% increase (*p* = 0.016) after exposure avoidance (correcting for treatment and baseline characteristics). Similarly, DLCO% decline of 0.23% per month was reversed to a DLCO% increase of 0.37% with an additional immediate increase of 4.0% after avoidance (*p* = 0.04). Although the 0.06% FVC% decline before avoidance reversed to a 0.28% monthly increase in fHP patients afterwards, these differences were not statistically significant (*p* = 0.15). DLCO% decline did not change in the fHP group (*p* = 0.54). Results are shown in Figure 6.

### 3.5. Clinical Behaviour Based on Symptoms Type and Chronicity

As the classical classification of HP patients into acute HP (AHP) and chronic HP (CHP) is also based on symptoms types (i.e. for AHP: systemic symptoms or temporal relation of symptoms with exposure, or both) and symptoms chronicity (i.e. for AHP: <6 months), we investigated whether these factors would influence survival and PFT evolution in the nfHP patients of the cohort. Neither symptom type (*p* = 0.482), nor symptoms chronicity (*p* = 0.187) had an effect on survival. Moreover both parameters did not affect FVC% evolution (symptom type: *p* = 0.80; symptom chronicity: *p* = 0.31) or DLCO% evolution (symptoms type: *p* = 0.55; symptoms chronicity: *p* = 0.11).

## 4. Discussion

Our study reports the effects of corticosteroid treatment and antigen avoidance in one of the largest HP cohorts published comprised of 202 HP patients that were divided according to their radiological phenotype (i.e. presence of fibrosis): 91 without fibrosis (nfHP) and 109 patients with fibrosis (fHP). We assessed the rate of FVC decline in nfHP and fHP (i.e. 0.93% and 0.56% per month, respectively). Although corticosteroids and exposure avoidance have been used for many years as the first line treatment of HP, this is the first study to assess the efficacy of these treatment options in both subgroups of this disease. Corticosteroid treatment was effective in nfHP concerning FVC% and DLCO% decline. In contrast, these treatments were not effective in fHP. Furthermore, we did not observe a survival impact of corticosteroid treatment in any subgroup. While exposure avoidance was significant with both FVC% and DLCO% decline reversed in nfHP and a positive trend in the FVC% decline in the fHP subgroup, no clear impact of exposure avoidance on survival was seen. Some observations deserve further attention. 

FVC% and DLCO% trajectories do not fully correlate with those presented by Salisbury [12] or Morisset [6]. Whereas in our data a monthly FVC% decline of 0.93% in nfHP and 0.56% in fHP was observed, Salisbury reported an FVC% increase of 1.92% in nfHP and a decline of 1.65% in fHP. Morisset reported a FVC% decline of 0.12% in chronic HP cases. However, we accounted for corticosteroid initiation and antigen avoidance in these trajectories which the abovementioned papers did not. Indeed, the differences in therapeutic effect of corticosteroid treatment we found between the two subgroups was remarkable. Presumably, as the mortality in nfHP was small, survival was not a good outcome measurement of treatment effect. PFT evolution, on the other hand, showed far better outcome measurement and clinically relevant effect sizes. In fHP, however, mortality was significant, but corticosteroids was ineffective. Moreover, no effect on PFT evolution was seen. These observations were supported with the results of Morisset [6], Adegunsoye [7], and Gimenez [22]. Finally, no interfering effects based on corticosteroid duration or dosage were seen. In order to assess whether the absence of therapeutic effect of fHP patients could be explained by a selective referral of fHP patients without a therapeutic effect, we analysed the subset of patients which was already followed at our centre before initiating corticosteroid therapy and showed similar results. These data underline the important medical need for new drugs to be tested in this patient group. 

Morisset et al. [6] determined the therapeutic effect of second line immunosuppressive treatment (i.e. Mycophenolate Mofetil and Azathioprine) and found an increase in DLCO% after treatment initiation in CHP patients. However, as it is not clear whether all CHP patients had established fibrosis, we presume that the treatment effect that was observed in the entire CHP cohort, could be driven by the non-fibrotic CHP patients. Moreover, Morisset et al. were also not able to find a therapeutic benefit for corticosteroid therapy. It is unclear whether an effect in their cohort would have been observed if the analysis was confined to only the non-fibrotic patients [6]. 

We observed worse outcome in HP patients without known antigen in multivariate analysis, which validates the data published by Fernandez-Perez et al. [11]. We further observed a better outcome in HP patients exposed to pigeons compared to moulds. This might be explained by a delay in diagnosing HP; as the exposure is more obvious in pigeon fanciers, diagnosis for HP in these patients would be easier for less experienced physicians [9]. As survival between mould exposed patients and patients without known exposure was similar, undiscovered mould exposure might be an important issue in patients without known exposure. 

Finally, as symptom chronicity and symptom type (classically more prevalent in AHP) did not alter clinical behaviour in nfHP patients, these results support the idea that a radiological stratification of HP patients is relevant.

There were several limitations to this study. First, being a retrospective study, the treatment effect of corticosteroid treatment with regard to survival could be biased as patients with more severe disease were more frequently treated with corticosteroids. We believe this should be taken into account when interpreting the trend to increased mortality in fHP patients treated with corticosteroids (HR 2.2, *p* = 0.096), as this trend was attenuated after correction for baseline disease severity in the multivariate analysis (HR 1.5, *p* = 0.46). Randomized controlled trials would be needed to further elucidate this question. Secondly, being an observational study of patients followed at a tertiary centre, there is a risk of referral bias, as already discussed regarding the lack of corticosteroid treatment effect in the fHP group. Thirdly, as a limited number of patients included in the cohort were treated with second line immunosuppressive drugs, the study lacked the necessary statistical power to assess the effect of this treatment option. As HP patients are not systematically treated with these therapies in many centres in Europe, this cohort might reflect this common practice in Europe.

## 5. Conclusions

In conclusion, we assessed the impact of corticosteroid treatment and antigen avoidance in a large cohort of 202 HP patients. Corticosteroid treatment increased FVC% and DLCO% in nfHP but not in fHP. Exposure avoidance was effective in nfHP, with a trend towards efficacy in fHP (*p* = 0.15). These data underpin the high medical need for new treatment options in fHP. 

## Figures and Tables

**Table 1:** Baseline characteristics for nfHP and fHP at diagnosis.

	nfHP (*N* = 93)	fHP (*N* = 109)	*p* Value
Age (year)	55.12 ± 13.19	65.29 ± 11.72	<0.001
Gender (male)	53 (57%)	69 (63.3%)	0.441
Ever smoker	36 (39.6%)	49 (45.8%)	0.46
Active smoker	1 (1.1%)	3 (3%)	0.624
Exposure unknown	10 (10.8%)	31 (28.4%)	0.003
Positive SsIgGs	53 (79.1%)	55 (63.2%)	0.06
BAL lymphocytosis	40.51 ± 25.57	19.64 ± 18.76	<0.001
FVC% baseline	81.61 ± 22.91	72.26 ± 21.89	0.005
DLCO% baseline	57.2 ± 20.7	45.3 ± 17.5	<0.001
Traction bronchiectasis	0 (0%)	87 (79.8%)	<0.001
Honeycombing	0 (0%)	40 (36.7%)	<0.001
Discussed at MDD	34 (39.1%)	61 (58.1%)	0.013
Corticosteroid treatment	67 (78.8%)	82 (80.4%)	0.934
2nd line immunosuppressive treatment *	8 (10%)	24 (24.5%)	0.021

Differences in demographic parameters, pulmonary function tests, specific IgG results and CT findings between nfHP and fHP patients. Data is presented as mean ± standard deviation (SD) or as patient numbers (%). * Azathioprine was initially used in all patients treated with second line immunosuppressive agent. Two fHP patients switched to methotrexate, one other to cyclophosphamide. One nfHP patient switched to mycophenolate. Definition of abbreviation: nfHP, non-fibrotic hypersensitivity pneumonitis; fHP, fibrotic hypersensitivity pneumonitis; SsIgGs, specific IgGs; BAL, broncho-alveolar lavage; FVC%, forced vital capacity (percent predicted); DLCO%, diffusing capacity of the lung for carbon monoxide (percent predicted); MDD, multidisciplinary discussion.

**Table 2:** **Frequency of exposure types based on HP subtype.** fHP patients had more often mould exposure and less often bird exposure, compared to nfHP patients. Percentages were calculated based on total patient counts of the HP subgroup.

	Birds	Mould	Other/Unclear	Unknown
nfHP	61 (65.6%)	16 (17.2%)	6 (6.4%)	10 (10.8%)
fHP	45 (41.3%)	24 (22.0%)	9 (8.3%)	31 (28.4%)

## Supplementary Materials

The following are available online at http://www.mdpi.com/2077-0383/8/1/14/s1, Appendix S1: Details on methods; Table S1: Report of MDD on cases not complying criteria for inclusion; Table S2: univariate and multivariate survival analyses; Table S3: Baseline characteristics according to corticosteroid treatment status; Table S4: Baseline characteristics according to 2nd line immunosuppressive treatment status; Figure S1: Inclusion of patients in the cohort, Figure S2: Survival in corticosteroid-treated patients, based on treatment duration, Figure S3: Survival in corticosteroid-treated patients, based on maximal dose of prednisolone-equivalent.
