# Supplementary material for: Effects of Corticosteroid Treatment and Antigen Avoidance in a Large Hypersensitivity Pneumonitis Cohort: A Single-Centre Cohort Study
Source: J Clin Med. 2018 Dec 21;8(1):14. doi: 10.3390/jcm8010014 (PMC6352061; doi:10.3390/jcm8010014)
Supplement: Supplementary file 1 [file jcm-08-00014-s001.pdf]

Supplementary Information

Title: Effects of corticosteroid treatment and antigen avoidance in a large Hypersensitivity Pneumonitis Cohort: a single-center cohort study.

Authors:

Laurens J De Sadeleer[1-2], Frederik Hermans[1], Els De Dycker[1], Jonas Yserbyt[1], Johny A Verschakelen[3], Eric K Verbeken[4], Geert M Verleden[1-2], Wim A Wuyts[1-2]

Affiliations:

1 Department of Respiratory Diseases, University Hospitals Leuven, Leuven, Belgium

2 Laboratory of Respiratory Diseases, KU Leuven, Leuven, Belgium

3 Department of Radiology, University Hospitals Leuven, Leuven, Belgium

4 Department of Pathology, University Hospitals Leuven, Leuven, Belgium

Corresponding Author:

Dr Laurens De Sadeleer

Department of Respiratory Diseases

University Hospitals Leuven

Herestraat 49

3000 Leuven

Belgium

laurens.desadeleer@kuleuven.be

Supplementary Appendix S1

Outcome

Patients were divided in 2 HP subgroups (nfHP and fHP), based on HRCT findings[9,12,13]: fibrosis was defined as the presence of reticulation, traction bronchiectasis and/or honeycombing on HRCT. HP patients without fibrosis on HRCT were included in the nfHP group, HP patients with fibrosis were included in the fHP group. Differences in baseline characteristics, survival and PFT evolution were assessed between the 2 groups.

Effect of corticosteroid therapy initiation on survival and FVC%/DLCO% decline was assessed. For survival analysis a binary parameter was used (ever- vs never-corticosteroid use). FVC% and DLCO% decline before corticosteroid initiation was compared to PFT decline after initiation. To minimize bias, PFT's after withdrawal of corticosteroid therapy were excluded. Survival analyses for corticosteroid dosage (maximal dosage of <40 mg vs >40 mg of prednisolone-equivalent) and duration of therapy (<6 months vs >6 months) was also performed.

Survival and PFT evolution regarding known/unknown exposure, exposure type (using binary parameter: birds vs mold) and exposure avoidance (using binary parameter: antigen avoided vs antigen not avoided) was assessed, comparable to corticosteroid treatment analysis. FVC% and DLCO% decline before antigen avoidance was compared to PFT decline afterwards.

PFTs 5 years before until 1 year after avoidance were used. Baseline characteristics, PFT evolution and survival of patients with known exposure were compared with patients without known exposure similarly to the analyses of the 2 HP subgroups. These analyses were also performed comparing different exposure types.

Statistical analysis

Baseline characteristics: continuous variables were analyzed using Student's t-tests and Mann-Whitney U-tests where appropriate. For discrete variables, chi-square tests and Fisher's exact tests were used, where appropriate. Patients who underwent lung transplantation were censored at the day of transplantation.

Survival analysis: outcome was based on 10-year survival. Data were displayed as Kaplan-Meier curves and analyzed using Cox proportional hazards models. In multivariate analyses, we corrected for age, gender and baseline FVC%. All multivariate analyses are shown in **Supplementary Table 1**.

PFT evolution: evolution of PFT was analyzed with linear mixed-effects models, using FVC% and DLCO% as outcome measurements (in separate analyses). Subject was corrected for as a random effect, both with random intercept and (independent) random slope. As PFT's were performed in both referring hospitals and the University Hospitals Leuven, the hospital where the PFT was performed

was also corrected for as random intercept. In general, time, age, gender were accounted for as fixed effect.

For analysis of the PFT evolution of the different HP subgroups, PFT's from the first year of follow-up were used when untreated and actively exposed.

For the corticosteroid treatment analysis: PFT's from 5 year before until 1 year after the treatment initiation were used. PFTs after stopping corticosteroid treatment (and/or stopping immunosuppression treatment) were excluded. Corticosteroid use was accounted for as fixed effect, both with and without time-varying covariate. Exposure status was corrected for as fixed effect, immunosuppression use was correct for as a time-varying covariate.

For the antigen avoidance analysis: PFT's from 5 year before until 1 year after avoidance were used. Exposure status as well as corticosteroid use was correct for with and without time-varying covariate, immunosuppression use was corrected for as time-varying covariate.

Patient number	Reason for not complying	Accepted at MDD	Reasoning for inclusion
1	Presenting without symptoms	yes	No symptoms but desaturation in rest. Bird exposure, suggestive CT pattern*, no biopsy performed
2	Presenting without symptoms	yes	Initial work-up after pneumonia with interstitial reticulation on chest X-ray. Bird exposure, suggestive CT pattern*, no biopsy performed
3	Presenting without symptoms	yes	Hypoxia diagnosed after GI investigation. Mould exposure, suggestive CT pattern*, no biopsy performed.
4	Presenting without symptoms	yes	Initial work-up after pneumonia with reticulonodular pattern on chest X-ray. Unknown exposure, BAL lymphocytosis 56%, suggestive CT pattern*, histopathology compatible with HP*.
5	Presenting without symptoms	yes	Interstitial abnormalities on chest X-ray, performed in the work-up of urticaria. Unknown exposure, BAL lymphocytosis 23%, suggestive CT pattern*, no biopsy performed
6	UIP-like CT pattern, no biopsy performed	yes	Mould exposure, limited peribronchovascular fibrosis, no clear apicobasal gradient of fibrosis
7	UIP-like CT pattern, no biopsy performed	yes	Unknown exposure but positive SsIgGs with Bal lymphocytosis 32%; limited ground glass opacities on chest CT
8	UIP-like CT pattern, no biopsy performed	yes	Bird exposure, also central and apical fibrosis on chest CT with limited ground glass opacities
9	UIP-like CT pattern, no biopsy performed	yes	Mould exposure, limited airtrapping on chest CT
10	Limited abnormalities on chest CT	yes	Occupational exposure with positive work resumption test. Limited ground glass opacities and air trapping, suggestive histopathology*

Supplementary Table S1: Report of MDD on cases not complying criteria for inclusion

Patients diagnosed with HP at the University Hospitals Leuven which did not comply with the criteria for validating the HP diagnosis were discussed in MDD. In the table both the reason for not complying, as well as the final decision and reasoning for inclusion were depicted.

*suggestive CT pattern, suggestive histopathological pattern: as described in the methods section of the main text. *Definition of abbreviation: MDD = multidisciplinary discussion, GI = gastro-intestinal, HP = hypersensitivity pneumonitis*

	Univariate			Multivariate		
	HR	CI	p-value	HR	CI	p-value
**nfHP vs fHP**	4.310	2.247 - 8.264	<0.001	2.247	1.112 - 4.545	0.024
**Corticosteroid use**	1.722	0.773 - 3.836	0.183	1.293	0.545 - 3.064	0.560
**Corticosteroid use in fHP patients**	2.228	0.867 - 5.727	0.096	1.961	0.711 - 5.410	0.194
**Known vs unknown exposure**	1.8	0.986 - 3.287	0.056	2.076	1.018 - 4.236	0.045
**Exposure avoidance**	1.235	0.554 - 2.755	0.606	1.289	0.567 - 2.932	0.544
**Bird exposure vs mould exposure**	2	0.979 - 4.155	0.057	2.8	1.322 - 6.05	0.007
**Bird exposure vs unknown exposure**	2.1	1.078 - 3.918	0.029	2.8	1.292 - 5.999	0.009

Supplementary Table S2: univariate and multivariate survival analyses

Results of cox proportional hazard models, both univariate and multivariate (correction for age, gender and baseline FVC%). For details of the specific groups that were analyzed, we kindly refer to the main text. *Definition of abbreviation: nfHP = non-fibrotic hypersensitivity pneumonitis, fHP = fibrotic chronic hypersensitivity pneumonitis*

	patients with corticosteroids	patients without corticosteroids	p-value
**Number of patients**	149	38	—
**Age (y)**	60.44 ± 13.62	60.09 ± 13.52	0.891
**Gender (male)**	82 (55%)	29 (76.3%)	0.028
**Ever smoker**	60 (40.5%)	20 (54.1%)	0.194
**Active smoker**	4 (2.8%)	0 (0%)	NA
**Exposure unknown**	22 (14.9%)	8 (21.1%)	0.498
**Positive SsIgGs**	82 (68.3%)	24 (82.8%)	0.333
**BAL lymphocytosis**	30.19 ± 25.55	25.82 ± 20.77	0.344
**FVC% baseline**	72.66 ± 21.73	91.46 ± 18.41	<0.001
**DLCO% baseline**	45.5 ± 16.5	65.09 ± 19.99	<0.001
**Fibrotic HP**	82 (55%)	20 (52.6%)	0.934
**Traction bronchiectasis**	68 (46%)	15 (39.5%)	0.594
**Honeycombing**	33 (22.3%)	4 (10.5%)	0.117
**Discussed at MDD**	69 (49.3%)	20 (54.1%)	0.741
**Initial corticosteroid dose (mg)***	40 (IQR 0)	—	—
**Duration of corticosteroid treatment (y) ***	0.54 (IQR 1.21)	—	—
**2nd line immunosuppressive treatment**	31 (22%)	1 (2.8%)	0.006

Supplementary Table S3: baseline characteristics according to corticosteroid treatment status

Differences in demographic parameters, pulmonary function tests, specific IgG results and CT findings between patients treated with corticosteroids and patients not treated with corticosteroids. Data is presented as mean ± standard deviation (SD) or as patient numbers (%). *As corticosteroid dose and treatment duration are severely skewed data, these were presented as median (inter quartile range (IQR)). *Definition of abbreviation: SsIgGs: specific IgGs, MDD = multidisciplinary discussion*

	patients treated with 2nd line immunosuppression	patients not treated with 2nd line immunosuppression	p-value
**Number of patients**	32	146	—
**Age (y)**	55.91 ± 14.07	61.36 ± 13.2	0.047
**Gender (male)**	19 (59.4%)	90 (61.6%)	0.969
**Ever smoker**	9 (28.1%)	69 (47.9%)	0.065
**Active smoker**	1 (3.2%)	3 (2.2%)	0.561
**Exposure unknown**	4 (12.5%)	26 (17.9%)	0.606
**Positive SsIgGs**	21 (77.8%)	79 (69.9%)	0.704
**BAL lymphocytosis**	24.57 ± 19.01	29.09 ± 25.3	0.392
**FVC% baseline**	68.59 ± 16.84	77.91 ± 23.22	0.034
**DLCO% baseline**	43.26 ± 16.88	51 ± 19.31	0.042
**Fibrotic HP**	24 (75%)	74 (50.7%)	0.021
**Traction bronchiectasis**	22 (68.8%)	59 (40.7%)	0.007
**Honeycombing**	10 (31.3%)	26 (17.9%)	0.147
**Discussed at MDD**	14 (48.3%)	68 (48.9%)	1
**Initial dose (mg)***	41 (IQR 13.7)	—	—
**Duration of treatment (y)***	0.44 (IQR 1.40)	—	—
**Corticosteroid treatment**	31 (96.9%)	110 (75.9%)	0.006

Supplementary Table S4: baseline characteristics according to 2nd line immunosuppressive treatment status

Differences in demographic parameters, pulmonary function tests, specific IgG results and CT findings between patients treated with 2nd line immunosuppression and patients not treated with 2nd line immunosuppression. Data is presented as mean ± standard deviation (SD) or as patient numbers (%). *As 2nd line immunosuppression dose and treatment duration are severely skewed data, these were presented as median (inter quartile range (IQR)). *Definition of abbreviation: SsIgGs: specific IgGs, MDD: multidisciplinary discussion*

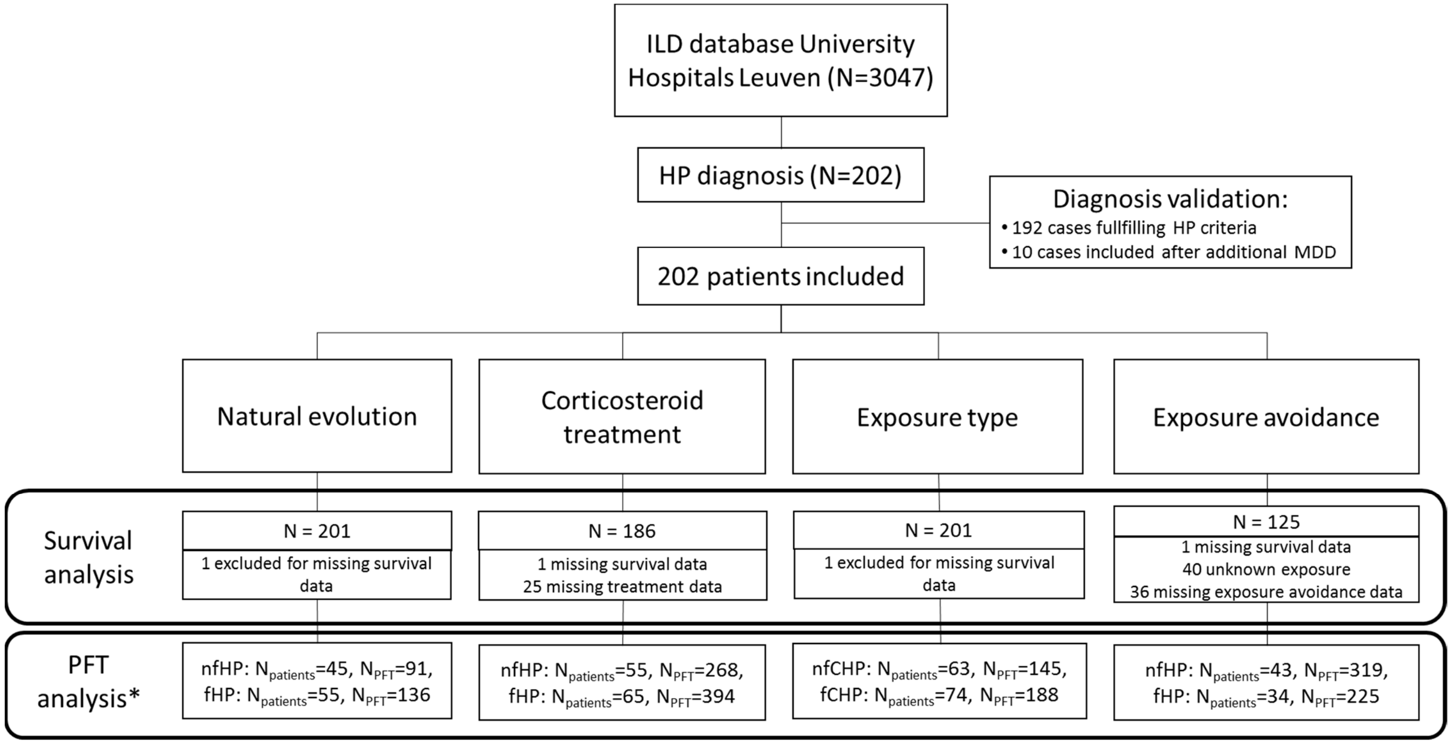

Supplementary Figure S1: Inclusion of patients in the cohort.

Visualization of cohort formation and exclusion rates for the different analyses. *all patients missing in the specific survival analysis were also excluded in the respective PFT analysis (e.g. 25 patients with missing treatment data were excluded from the PFT analysis of corticosteroid treatment), apart from one patient which lacked survival data (excluded from the survival analysis but included in the PFT analysis). Other patients excluded from the PFT analysis were missing longitudinal PFT data. D*efinition of abbreviation: ILD = interstitial lung disease, HP diagnosis = Hypersensitivity pneumonitis diagnosis, MDD = multidisciplinary discussion, nfHP = non-fibrotic hypersensitivity pneumonitis, fHP = fibrotic chronic hypersensitivity pneumonitis, PFT analysis = pulmonary function test analysis,* $N_{patients}$ *= number of patients included in the analysis,* N_{PFT} *= number of pulmonary function tests included in the analysis.*

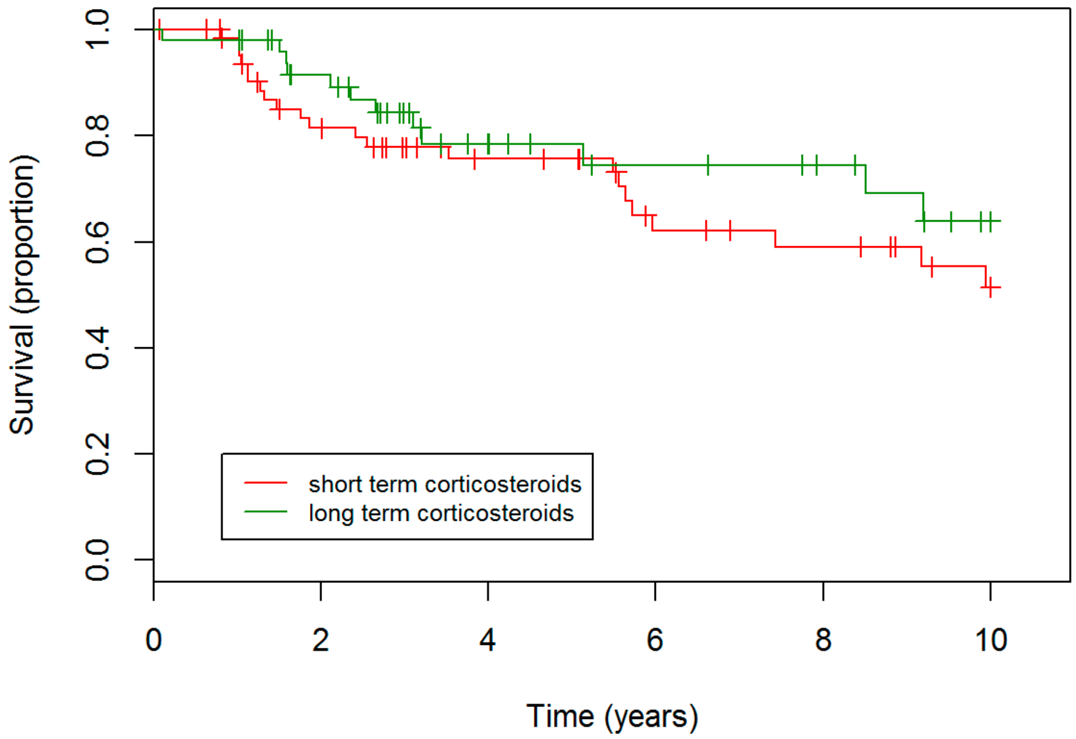

Supplementary Figure S2. Survival in corticosteroid-treated patients, based on treatment duration. Short term (N=51) was determined as < 6 months, long term (N=51) was determined as > 6 months. No statistically significant differences were observed.

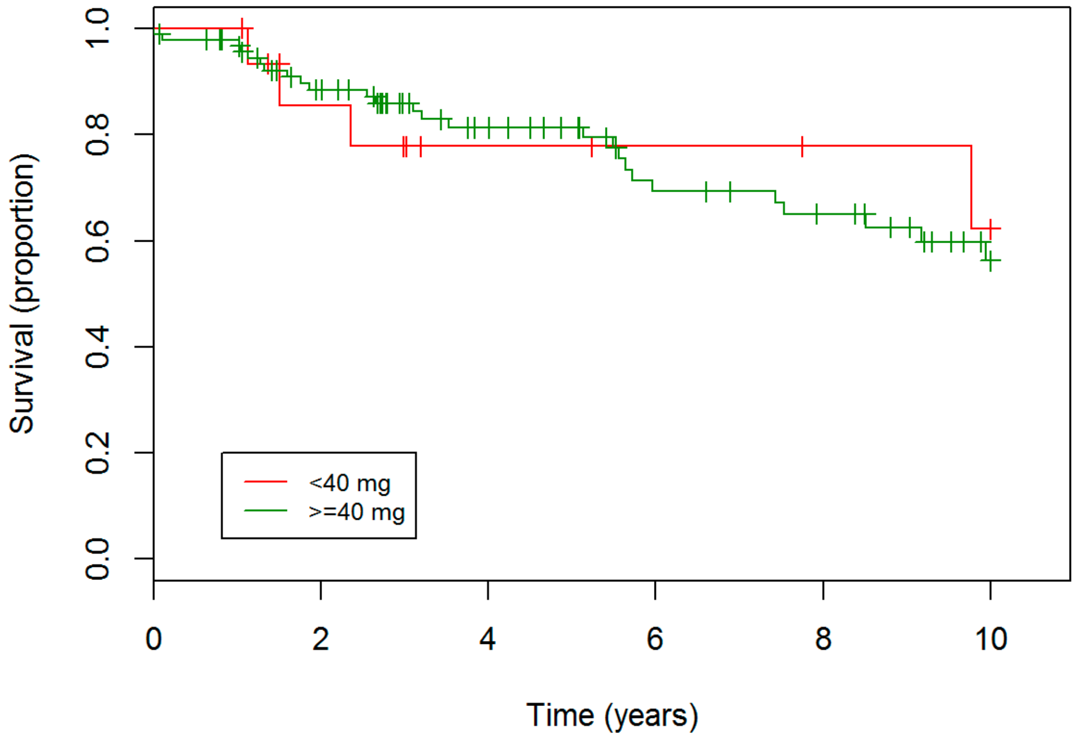

Supplementary Figure S3. Survival in corticosteroid-treated patients, based on maximal dose of prednisolone-equivalent.

No statistically significant differences were observed between low dose-treated patients (N=16) and high-dose treated patients (N=94).
